# Electroacupuncture for Temporomandibular Disorders: A Systematic Review of Randomized Controlled Trials

**DOI:** 10.3390/healthcare9111497

**Published:** 2021-11-02

**Authors:** Soo-Hyun Sung, Dongsu Kim, Minjung Park, Su-In Hwang, Young-Jin Yoon, Jang-Kyung Park, Hyun-Kyung Sung

**Affiliations:** 1 Department of Policy Development, National Institute of Korean Medicine Development, Seoul 04554, Korea; koyote10010@nikom.or.kr; 2 College of Oriental Medicine, Dongshin University, Naju-si 58245, Korea; dskim20@dsu.ac.kr; 3 Center for Development of Innovative Technologies in Korean Medicine, National Institute of Korean Medicine Development, Seoul 04554, Korea; mj.park@nikom.or.kr; 4 Department of Korean Medicine Obstetrics and Gynecology, School of Korean Medicine, Pusan National University, Yangsan 50612, Korea; hwangsi1216@gmail.com (S.-I.H.); yyj@pusan.ac.kr (Y.-J.Y.); 5 Department of Korean Medicine Pediatrics, School of Korean Medicine, Semyung University, Jecheon 27136, Korea

**Keywords:** electroacupuncture, electroacupuncture therapy, temporomandibular disorders, systemic review, randomized controlled trial, complementary and alternative medicine

## Abstract

Although electroacupuncture (EA) is an effective treatment for pain relief, there has been no systematic review of EA treatment for temporomandibular disorder TMD. This systematic review aimed to evaluate the efficacy and safety of EA in TMD management. We searched 14 databases until April 2021 for randomized controlled trials (RCTs) evaluating the effects of EA on TMDs. Eleven RCTs with 667 patients that used three acupuncture points (ST6, ST7, and LI4) were included. Two RCTs reported significant effects of EA plus microwave treatment compared with EA treatment alone on the total effectiveness rate (TER) for TMD. Further, two studies reported that compared with ultrashort wave alone, EA plus ultrashort wave had a significant effect on the TER for TMD and visual analog scale. All RCTs did not report adverse events. Our findings demonstrated the positive potential of EA in TMD management. However, there was weak evidence regarding EA use for TMD management given the poor quality and small sample sizes of the included studies. In the future, well-designed RCTs are required. It is necessary to investigate clinical trials and systematic reviews to compare the effectiveness and safety of EA and acupuncture for TMD.

## 1. Introduction

Temporomandibular disorders (TMDs) is a generic term for various symptoms caused by abnormalities in the temporomandibular joint (TMJ), surrounding muscles, and related tissues [1,2]. Symptoms and signs of TMDs include a limited or deviating range of motion, clicking with function, TMJ pain, pain on jaw opening, orofacial pain, and headache [1,3,4]. TMDs are diagnosed by Axis Ⅰ, derived from clinical signs and symptoms, and Axis Ⅱ associated with psychosocial and behavioral status [5].

The TMJ is among the most frequently used body joints; therefore, TMD is a very common condition [6,7]. The reported prevalence of TMD varies widely across different studies, with most studies reporting that 40% to 60% of the population had one or more signs and 25% complained of pain [8,9]. The disease is common among individuals aged 20–40 years. Additionally, the prevalence in females is twice that in males, which could be attributed to female hormones [8,10].

Recent studies have demonstrated that TMD has a multifactorial etiology including anatomical, pathological, physiological, social, and mental causes, as well as personality traits and history of trauma [4,11,12,13]. TMD may be associated with other chronic pain, anxiety, and depression; therefore early TMD treatment is crucial [8,14]. Treatments for TMD include conservative treatments, including medication, physical therapy, and patient education, as well as invasive treatments such as surgery. In most cases, non-invasive treatment can improve the TMD symptoms. A recent study recommended surgery only when nonsurgical treatment cannot improve symptoms [4,12].

Raphael et al. [15] and DeBar et al. [16] reported that 22.2% to 62.5% and 4.8% to 15.9% of all patients with TMD in the United States undergo treatment using complementary and alternative medicine and acupuncture, respectively. There have been systematic reviews on acupuncture treatment of TMD [17,18,19]. Compared with sham acupuncture, acupuncture is more effective against pain [17,18,19]. Electroacupuncture (EA), which combines manual acupuncture with an electric stimulus, is a common treatment method for musculoskeletal diseases and injuries and is more effective for pain relief than manual acupuncture [20,21]. However, there has been no systematic review of EA treatment for TMD. This systematic review aimed to summarize the results of randomized controlled trials (RCTs) to evaluate the clinical evidence regarding the efficacy and safety of EA in patients with TMD. Examining the applicability of EA from the perspective of evidence-based medicine potentially improves the management of TMD, thereby contributing to recommending EA for TMD as an evidence-based treatment.

## 2. Materials and Methods

### 2.1. Protocol and Registration

Our systematic review protocol was registered in the international prospective register of systematic reviews under the registration number PROSPERO 2021: CRD42021229712 (available from: https://www.crd.york.ac.uk/prospero/display_record.php?RecordID=229712, accessed on 23 March 2021).

### 2.2. Data Sources and Searches

We searched PubMed, MEDLINE, EMBASE, Physiotherapy Evidence Database, Cochrane Central Register of Controlled Trials, and CINAHL Plus electronic databases for articles published up to April 2021. Further, we searched six Korean databases (Korea Institute of Science and Technology Information, Korean Traditional Knowledge Portal, KoreaMed, OASIS, RISS, and the National Library of Korea) and two Chinese databases (CNKI and WangFang). There were no language-based limitations regarding the papers chosen for our review.

The search terms were as follows: “temporomandibular joint disorder OR temporomandibular disorder OR temporomandibular joint disease OR temporomandibular disease OR jaw disease” AND “electroacupuncture OR electric acupuncture OR electrical acupuncture” AND “randomized controlled trial OR randomized clinical trial”.

### 2.3. Study Selection

#### 2.3.1. Types of Studies

We included all RCTs that evaluated the effect of EA on TMD. We excluded non-randomized trials, including clinical studies (case studies, case series, and case-controlled trials), animal studies, experimental studies, surveys, and reviews.

#### 2.3.2. Participants

We included all patients diagnosed with TMD without age- or sex-based restrictions.

#### 2.3.3. Types of Interventions

We included all types of EA used for TMD treatment. EA was defined by the current flow from connecting the electro-stimulator after inserting the acupuncture needles into the acupoints [21].

#### 2.3.4. Types of Comparisons

We compared EA with no treatment, placebo/sham treatment, or conventional treatment. Further, we included RCTs comparing EA with EA plus conventional treatment and EA plus conventional treatment with identical conventional treatment. We excluded unqualified control interventions (Chinese manipulation) given their unproven efficacy. 

#### 2.3.5. Types of Outcome Measures

In this study, we considered the pain score, functionality score, and effectiveness rate for TMD as primary outcomes. The effective rate was defined as the number of patients who showed improvement in visual acuity and retinal vascular-related abnormalities. Secondary outcomes included quality of life, activity scores, and adverse events.

### 2.4. Data Extraction

Three authors (S.-H. Sung, M. Park, and S.-I. Hwang) independently extracted data using a predefined data extraction form. Further, two independent reviewers (S.-H. Sung and Y.-J. Yoon) collected data regarding author information, sample size, interventions, treatment sessions, outcome measures, main results, and adverse events. Regarding the EA interventions, we extracted the following data: regimen, acupuncture points, needle type, depth of insertion, angle of insertion, needle retention time, and frequency of electric stimulation. In case of insufficient outcome data, the corresponding authors were contacted whenever possible. Disagreements were resolved through discussion with J.-K. Park.

### 2.5. Assessment of Risk of Bias (ROB)

Two independent researchers (M. Park and Y.-J. Yoon) evaluated the ROB for the included RCTs based on the Cochrane Collaboration’s ROB tool [22]. The Cochrane Collaboration’s tool comprises seven domains; however, we assessed the following six evaluation methods: (1) random sequence generation; (2) allocation concealment; (3) blinding of participants; (4) blinding of assessors; (5) incomplete outcome data; and (6) selective outcome reporting. For each domain, the ROB was rated as low risk (L), high risk (H), or unclear (U). Disagreements in scoring were resolved through discussions with J.-K. Park.

### 2.6. Data Analyses

Statistical analyses were performed using RevMan 5.3 (version 5.3 for Windows; the Nordic Cochrane Centre, Copenhagen, Denmark). Continuous and dichotomous data were expressed as mean differences and risk ratios, respectively, with 95% confidence intervals. The I^2^ test was used to assess among-study heterogeneity, with I^2^ values of 0–40%, 30–60%, 50–90%, and 75–100% representing no/mild, moderate, substantial, and full heterogeneity, respectively [23]. Fixed and random effect models were used when the I^2^ value was <50% and >50%, respectively, with subgroup analysis being conducted to identify the possible heterogeneity causes [23]. Sensitivity analysis was planned using trials of low ROB for examining the possible contribution of methodological quality. A summary of the findings is discussed in the results in case meta-analysis was not possible due to the considerable variation in the study characteristics.

## 3. Results

### 3.1. Study Selection and Description

**Figure 1:**
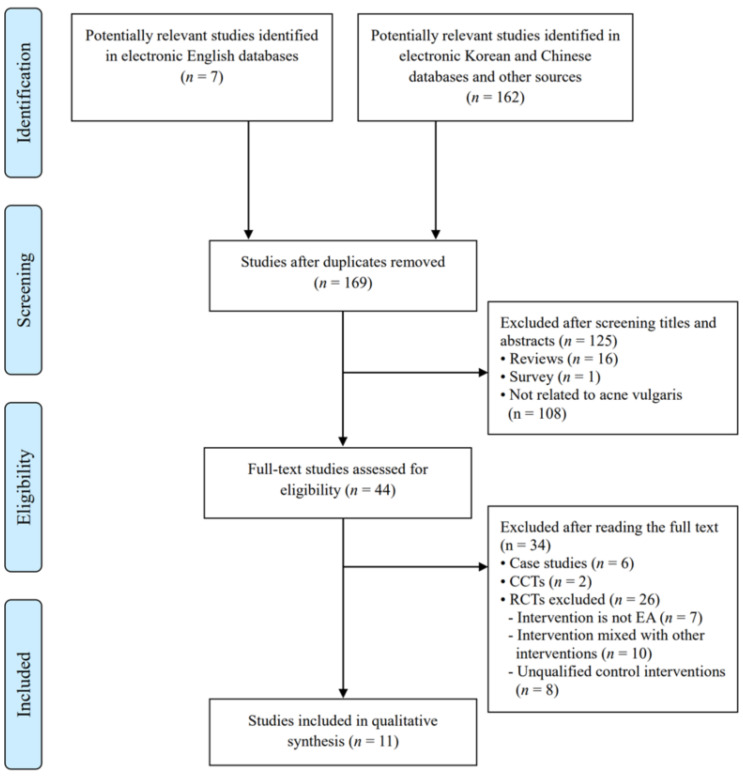
Flowchart of the RCT selection process. CCTs, controlled clinical trials; EA, electroacupuncture; RCTs, randomized controlled trials; TMD, temporomandibular disorders.

The database queries identified 169 potentially relevant studies; among them, we included 11 RCTs (English databases: *n* = 1, Chinese databases: *n* = 10). All studies [24,25,26,27,28,29,30,31,32,33,34] were conducted in China and published in Chinese. One RCT [28] was published in Chinese journals and indexed in the English databases. Figure 1 shows a flow chart of the study selection process as recommended in the Preferred Reporting Items for Systematic Reviews and Meta-Analyses guidelines [35]. Table 1 summarizes the details of the included studies. 

### 3.2. Participants

We included 667 patients with TMD (263 men, 404 women). The experimental and control groups included 331 and 336 patients, respectively. In the final analyses, 331 and 326 patients from the experimental and control groups, respectively, were included. 

### 3.3. Intervention

We compared EA treatment with block therapy (injection) [25,29] or EA plus microwave [26,30] or physiotherapy [27] or acupuncture [31]. Two RCTs compared EA plus ultrashort wave with ultrashort wave only [33,34]. One study separately compared EA treatment with EA plus ultrashort wave [24], EA plus massage therapy [28], and EA plus extracorporeal shock wave [32]. Table 2 shows the characteristics of the EA treatment in the included RCTs.

#### 3.3.1. EA Points

**Figure 2:**
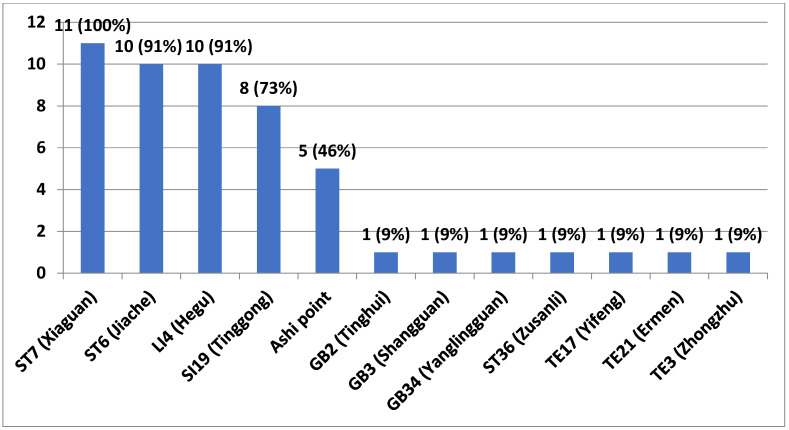
The acupuncture points utilized in these studies.

Ten studies used 11 different acupuncture points, with each study using an average of 5 (4–7) acupuncture points. All 11 studies used the acupuncture points of Jiache (ST6), Xiaguan (ST7), and Hegu (LI4) [24,25,26,27,28,29,30,31,32,33,34]. Further, Tinggon (SI19) was used in seven studies [24,27,28,29,32,33,34] while Ashi point was used in five studies [25,26,30,32,33]. Tinghui (GB2) [29], Shangguan (GB3) [30], Yanglingquan (GB34) [33], Zusanli (ST36) [34], Yifeng (TE17) [33], and Ermen (TE21) [29] were each used in one study (Figure 2).

#### 3.3.2. Needle Type (Diameter, Length)

The needle length used in EA treatment was specified in the 11 studies [24,25,26,27,28,29,30,31,32,33,34]. Eight studies [24,25,26,27,28,29,30,32] used needles of a length of 40 mm while one study [33] used needles of two different lengths (25 and 40 mm). The remaining studies used needles with a length of 25 mm [31,34].

Eight studies [24,25,26,28,30,32,33,34] used needles of three different diameters (0.25, 0.32, and 0.35 mm); among them, five studies [25,26,30,33,34] used a needle with a diameter of 0.25 mm. One study [24] used a needle with a diameter of 0.35 mm, and one study [31] used a needle with a diameter of 0.30 mm. Two studies did not report the diameter of the needles [27,29].

#### 3.3.3. Depth of Insertion

Three RCTs [24,26,30] reported the depth of insertion. Among them [26,30], one acupuncture point (ST7) was acupunctured to a depth of 25–30 mm, while another study [29], deployed the needle to a depth of 0.5–1.5 inches.

#### 3.3.4. Angle of Insertion

Three studies reported a 90° (perpendicular to the skin) angle of insertion [24,26,30].

#### 3.3.5. Needle Retention Time

The needle retention time was 15–30 min. The retention time was 30, 20, and 15–20 min in seven [24,25,26,30,31,32,33], three [28,29,31], and one study [27], respectively. 

#### 3.3.6. Frequency of Electric Stimulation

The frequencies used in the EA treatment ranged from 1.2 Hz to 50 Hz. The frequency used was 1.2 Hz in two studies [28,31] and 50 Hz in two studies [26,30]. Further, one study used a frequency of 2 Hz [24] and 20 Hz [31] while another used a frequency of 1.45 Hz [32]. One study [33] reported an unspecified low frequency. The other three studies [25,27,29] did not report the frequency of EA treatment.

### 3.4. Outcomes

The included studies reported 11 outcome measures. Regarding the total effectiveness rate (TER) for TMD, three to five symptoms were comprehensively evaluated to measure the effect. Table 3 summarizes the detailed measurement method of outcome TER for TMD. 

#### 3.4.1. EA versus Block Therapy

Two RCTs [25,29] compared EA treatment with block therapy. One RCT [25] reported significant effects in the EA group compared to the block therapy group, with respect to the total effectiveness rate (TER) for TMD (*p* < 0.05). Another study [29] that compared EA treatment to block therapy showed significant efficacy in the control group with respect to the TER for TMD (*p* < 0.05).

#### 3.4.2. EA versus EA plus Microwave

Regarding the primary outcome TER for TMD, two studies [26,30] showed significant effectiveness with treatment by EA plus microwave compared with treatment by EA only (*p* < 0.05).

#### 3.4.3. EA plus Ultrashort Wave versus Ultrashort Wave Only

A meta-analysis could not be conducted due to variations in outcome measures in two RCTs [33,34] that compared EA plus ultrashort wave with ultrashort wave only. Compared with ultrashort wave only, EA plus ultrashort wave had a significantly better effect with respect to TER for TMD (*p* < 0.05) [33] and visual analog scale (VAS; *p* < 0.01) [34]. Hu. et al. [33] reported a significant between-group difference in the secondary outcome of relapse rate. One study [34] reported that compared with ultrashort wave, EA plus ultrashort wave significantly improved the secondary outcome “painless opening degree” (*p* < 0.01). 

#### 3.4.4. EA versus EA plus Ultrashort Wave

In one study, treatment with EA and ultrashort wave showed significant effectiveness in improving TER for TMD compared to EA treatment alone (*p* < 0.01) [24].

#### 3.4.5. EA versus Physiotherapy

Compared with EA treatment, physiotherapy had significant effects on TER for TMD (*p* < 0.01) [27].

#### 3.4.6. EA versus EA plus Massage Therapy

An RCT conducted by Bu et al. [28] evaluated the effect of EA on patients with TMD. EA treatment plus massage therapy significantly improved the TER for TMD (*p* < 0.05), myofascial pain (*p* < 0.05), and external pterygoid muscle spasm (*p* < 0.05).

#### 3.4.7. EA versus Acupuncture

Compared with traditional acupuncture, EA had a significant better effect in improving TER for TMD (*p* < 0.05) and visual analog scale (VAS; *p* < 0.05) [31].

#### 3.4.8. EA versus EA plus Extracorporeal Shock Wave

One study [32] reported that compared with EA alone, EA treatment plus extracorporeal shock wave significantly improved the VAS score (*p* < 0.05), TER for TMD (*p* < 0.05), maximum opening degree (*p* < 0.05), and Fricton’s TMJ Dysfunction Index (*p* < 0.05).

### 3.5. Adverse Events

All included RCTs did not mention adverse events.

### 3.6. Assessment for ROB

Table 4 summarizes the details of the ROB for each RCT. Regarding the randomization procedure, only one study [33] reported an appropriate randomization procedure using a computer random number generator. Further, two studies [24,34] were considered to have high ROB, as they assigned participants to treatment groups based on the treatment order. The remaining seven studies [25,26,27,28,29,30,31,32] did not mention random sequence generation. None of the included clinical trials reported the allocation concealment method. Given the different intervention types administered to the groups, all studies did not perform blinding of participants. Except for one RCT, the remaining RCTs [25,26,27,28,29,30,31,32,33,34] did not describe the blinding of outcome assessment. In one study, patients with TMD were evaluated by a third assessor [24]. There were no missing data in ten studies [24,26,27,28,29,30,31,32,33,34]. One RCT [25] had missing data; however, the dropout rate was ≤20% with a short-term follow-up. No trial provided information regarding the published or registered study protocols.

### 3.7. Publication Bias

**Figure 3:**
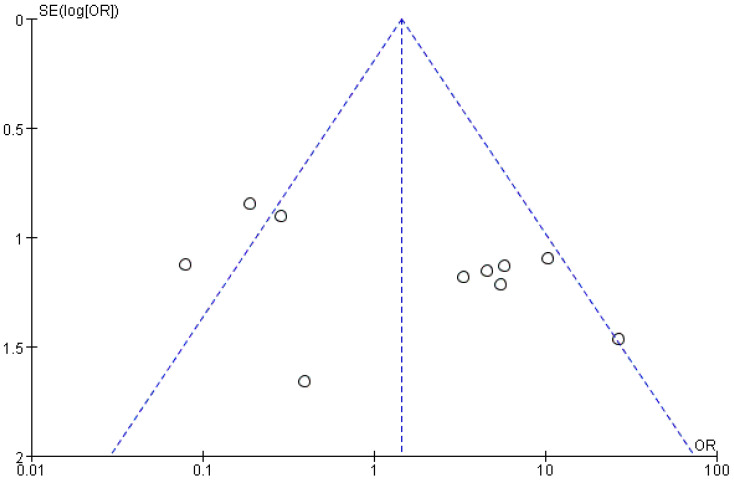
Funnel plot: TER for TMD.

A funnel plot of the primary outcome (TER for TMD) was constructed. There was no significant asymmetry seen in the visual inspection of the funnel plot (Figure 3).

## 4. Discussion

TMD is among the most common causes of non-dental pain in the orofacial region. It is characterized by frequent relapses and is correlated with other types of chronic pain. As symptoms can be mitigated with proper treatment, prompt TMD treatment is essential. In most cases, non-invasive treatments, including medication, physical therapy, and prostheses, are used [8,12,14,37].

Non-steroidal anti-inflammatory drugs (NSAIDs) are among the most widely used conservative treatments [13]. However, given their side effects, including gastric erosion and ulcers, NSAIDs are contraindicated in patients with active gastrointestinal diseases.

Furthermore, the efficacy of NSAIDs can be compromised if they are concomitantly administered with angiotensin-converting enzyme inhibitors. Additionally, if they are concomitantly administered with warfarin, there is an increased risk of hemorrhagic complications. Therefore, in some cases, it is difficult to use NSAIDs in conjunction with existing medical drugs [2]. Physiotherapy, which relieves the symptoms of neuromuscular diseases, is among the most commonly used conservative treatments. An ultrashort wave has the ability to expand topical blood vessels to increase blood flow and effectively regenerate microwave peripheral nerve damage [38,39]. A massage may stimulate mechanoreceptors within muscular and connective tissue to reduce pain in the muscles and joints [40]. Further, an extracorporeal shock wave exerts analgesic effects, which promote soft tissue remodeling and repair [41]. 

In traditional Chinese medicine, acupuncture is widely used in numerous musculoskeletal disorders, as it can induce analgesic effects with minimal and minor side effects. Specifically, EA treatment, which involves both acupuncture and electrical stimuli, yields better analgesic effects [20,21]. 

A systemic review of patients with tension-type headache, which is a myofascial pain, revealed that compared with physical therapy alone, physical therapy combined with acupuncture has a better relieving effect [42]. 

We analyzed 11 RCTs on the efficacy of EA in patients with TMD. Generally, EA with conventional treatment [24,26,28,30,32,33,34] was more effective than treatment with EA alone. However, there were very few RCTs; moreover, control intervention types were too varied to draw definite conclusions. Therefore, our results should be interpreted carefully.

In all included studies, the three most commonly used acupuncture points for treating TMD were ST6, ST7, and LI4 [34,43,44]. Jiache (ST6) and Xiaguan (ST7) are located in the TMJ; therefore, they ensure more active Qi circulation in patients with TMD. Hegu (LI4) is located in the lateral elbow and is related to systemic balancing using muscle relaxation [45]. Muscles surrounding the TMJ remain tense due to food chewing, stress, and tense neck muscles [46], which could explain the muscle relation effects of EA on Hegu (LI4).

Although we did not focus on the side effects, the number of participants analyzed and randomized was the same, which suggests that there were no significant side effects leading to withdrawals from the studies. 

To our knowledge, this is the first systematic review of EA treatment effects on the TMJ. We compared conventional treatments combined with EA with EA treatment alone. Additionally, we screened study papers published in English, Korean, and Chinese without limiting the search to any particular language. 

Most of the included RCTs had unclear ROBs for proper randomization, allocation concealment, blinding of outcome assessment, and selective reporting. Only one study had both appropriate randomization [33] and adequate blinding for outcome assessment [24]. Therefore, there is limited validity of the statistical analysis of the included studies. There is a need for more randomized, double-blinded, multi-center clinical trials that are well designed with rigorous methodology. Second, based on the Standards for Reporting Interventions in Clinical Trials of Acupuncture (STRICTA) guidelines, the following factors affect the efficacy of EA treatment: EA points; needle type; depth of insertion; frequency and intensity of electric stimulation; and number, frequency, and duration of treatment sessions [47]. Among the 11 studies, only 2 studies had the same EA intervention characteristics [26,30]. Moreover, eight studies did not report the depth of insertion [24,25,27,28,31,32,33,34]; further, eight studies did not report the angle of insertion [25,27,28,29,31,32,33,34]. Two studies did not report the needle diameter [27,29], which impedes the assessment of factors that could influence the effect. Third, the most common treatment and the evaluation tools varied, which impeded meta-analysis. Systemic reviews and meta-analyses measure treatment efficacy by synthesizing the study data [48]. There is a need for further clinical trials to verify the treatment effects of EA. Fourth, there were heterogeneous outcomes of TER for TMD used in the included RCTs. Given the variation of symptoms for evaluating the effectiveness rate, it was difficult to perform a meta-analysis. Finally, we included only one paper that set the control group as acupuncture treatment in this systematic review. Estimating through the funnel plot, I think there will be some publication bias. However, as there are few studies included, so further analysis will be needed later. In the future, it is necessary to strengthen the level of clinical evidence of EA and acupuncture of TMD, and accordingly, updated systematic review of EA for TMD is required.

Following elements should be standardized based on the STRICTA guidelines [46]: diameter and length of needle, depth and angle of injection, number of treatment sessions, needle retention time, and electric stimulation. Our findings confirmed the potential applicability of EA in TMD treatment. EA interventions and outcome measures have not been standardized. Based on this review, the following treatment regimens are suggested for future larger scale RCTs: (1) ST6, ST7, LI4 and SI19 for EA points, (2) 40 nm for needle length, (3) 25–30 mm for depth of insertion, (4) 90° for angle of insertion, (5) 15–30 min for needle retention time, (6) 1.2 to 50 Hz for frequency of electric stimulation. Control groups should be sham EA treatments or no treatment to clarify the efficacy of EA. Additionally, there is a need to research the standard evaluation tools for TMD evaluation. Specifically, the evaluation standard for the outcome TER for TMD should be the same to facilitate the evaluation of the clinical effectiveness.

## 5. Conclusions

Our findings demonstrated that EA treatment was more effective in combination with other interventions than when conducted alone. However, in the included RCTs there was low methodological quality, the interventions and outcome measures used were heterogeneous, and side effects were not mentioned. To recommend EA for treating TMD in routine clinical practice, well-designed, high quality, and multi-center RCTS are needed. Additionally, in clinical settings, acupuncture treatment was more widely used for treating TMD than EA. In the future, it is necessary to investigate clinical trials and systematic reviews to compare the effectiveness and safety of EA and acupuncture for TMD.

## Figures and Tables

**Table 1:** Characteristics of the included studies.

Author, Year	Sample Size (m/f)	Experimental Group (No. of Participants Analyzed/Randomized)	Control Group (No. of Participants Analyzed/Randomized)	Outcome Measures	Main Results	AE
Liu (2007) [24]	62 (11/51)	EA (31/31)	EA + Ultrashort wave ^a1^ (31/31)	TER for TMD	Negative ^i^	n.r.
Wang (2009) [25]	96 (46/50)	EA (48/48)	Block therapy (38/48) ^b1^	TER for TMD	Positive ^h^	n.r.
Jia (2010) [26]	35 (15/20)	EA (14/14)	EA + Microwave (21/21) ^c1^	TER for TMD	Negative ^h^	n.r.
Liu (2010) [27]	51 (28/23)	EA (26/26)	Physiotherapy (25/25) ^d^	TER for TMD	Negative ^i^	n.r.
Bu(2011) [28]	96 (30/66)	EA (48/48)	EA + Massage therapy (48/48) ^e^	(1) TER for TMD	(1) Negative ^h^	n.r.
				(2) TER for myofascial pain	(2) Negative ^h^	
				(3) TER for external pterygoid muscle spasm	(3) Negative ^h^	
Li (2011) [29]	54 (12/42)	EA (27/27)	Block therapy (27/27) ^b2^	TER for TMD	Negative ^h^	n.r.
Chen (2012) [30]	64 (25/39)	EA (32/32)	EA + Microwave (32/32) ^c2^	TER for TMD	Negative ^h^	n.r.
Zhang (2014) [31]	60 (26/34)	EA (30/30)	Acupuncture (30/30) ^f^	(1) VAS	(1) Positive ^h^	n.r
				(2) TER for TMD	(2) Positive ^h^	
Han (2018) [32]	40 (23/17)	EA (20/20)	EA+ Extracorporeal Shock Wave (20/20) ^g^	(1) VAS	(1) Negative ^h^	n.r.
				(2) TER for TMD	(2) Negative ^h^	
				(3) Maximum opening degree	(3) Negative ^h^	
				(4) Fricton’s TMJ Dysfunction Index	(4) Negative ^h^	
Hu (2018) [33]	69 (28/41)	EA + Ultrashort wave (35/35)	Ultrashort wave (34/34) ^a2^	(1) TER for TMD	(1) Positive ^h^	n.r.
				(2) Relapse rate (after 3 months)	(2) Positive ^h^	
Ye (2019) [34]	40 (19/21)	EA + Ultrashort wave (20/20)	Ultrashort wave (20/20) ^a3^	(1) VAS	(1) Positive ^i^	n.r.
				(2) Painless opening degree	(2) Positive ^i^	

^(a1–3)^ Ultrashort wave therapy: one of the thermal therapy methods with high frequency (mainly wavelength 6–8 m) current. ^(a1)^ 50–80 mA, 20 min, ^(a2)^ 80 mA, 40 W, 43 MHz, 15 min ^(a3)^ 40.68 MHz, wavelength 7.37 m, 20 min. ^(b1–2)^ Block therapy: to achieve anti-inflammatory and analgesic purposes by injecting a mixture of local anesthetics and hormones into the pain area. ^(b1)^ Injection (triamcinolone acetonide 40 mg, 2% lidocaine) ^(b2)^ Injection (lidocaine, hydrochloride, prednisolone acetate, mixing 1 mL of each). ^(c1–2)^ Microwave therapy: electrical therapy using electromagnetic waves of 1 m or less and 1 mm or more than 1 mm (mainly using 2450 MHz). ^(c1)^ 2450 MHz, AC 220 V/50 Hz. 15 min, ^(c2)^ 2450 MHz, AC 220 V/50 Hz. 15 min. ^(d)^ Physiotherapy: combination of TDP lamp therapy, electrotherapy (medium frequency, 5.0–8.0 mA) and ultrashort wave therapy (50 mA). ^(e)^ Massage therapy: finger massage to GB3, ST7, ST6, SI19 and manipulation of jaw joint. ^(f)^ Acupuncture: TE3, ST7, SI19 (0.30 mm, 25 mm). ^(g)^ Extracorporeal shock wave therapy: shock wave therapy is a non-invasive method that uses pressure waves to treat various musculoskeletal conditions using Dolorclastmaster (7 Hz, 15 mm, 2000 times). ^(h)^ *p* < 0.05; ^(i)^ *p* < 0.01. AE, adverse event; EA, electroacupuncture; F, female; M, male; No., number; n.r., not reported; TER, total effectiveness rate; TMD, temporomandibular disorder; TMJ, temporomandibular joint; VAS, visual analog scale.

**Table 2:** Characteristics of electroacupuncture interventions in the included studies.

Author, Year	Regimen	Acupuncture Points	Type of Needle (Diameter, Length)	Depth of Insertion	Angle of Insertion	Needle Retention Time	Frequency of Electric Stimulation	Co-Interventions
Liu (2007) [24]	20 sessions (20 days)	ST7, ST6, SI19, LI4	0.35 mm, 40 mm	n.r.	90°	30 min	2 Hz	Ultrashort wave
Wang (2009) [25]	14 sessions (14 days)	ST7, ST6, LI4, Ashi point *	0.25 mm, 40 mm	n.r.	n.r.	30 min	n.r	None
Jia (2010) [26]	10 sessions (once a day for 10 days)	ST7, ST6, LI4, Ashi point *	0.25 mm, 40 mm	ST7: 25–30 mm Etc: n.r.	ST7: 90° Etc: n.r.	30 min	50 Hz	Microwave
Liu (2010) [27]	10 sessions (10 days)	LI4, ST7, ST6, SI19	n.r., 40 mm	n.r.	n.r.	15–20 min	n.r.	None
Bu(2011) [28]	40 sessions (40 days)	GB3, ST7, ST6, SI19, LI4	0.32 mm, 40 mm	n.r.	n.r.	20 min	1.2 Hz	Massage therapy
Li (2011) [29]	5 sessions (every other day for 10 days)	TE21, ST6, ST7, LI4, GB2, SI19	n.r., 40 mm	0.5–1.5 inch	n.r.	20 min	n.r.	None
Chen (2012) [30]	10 sessions (10 days)	ST7, ST6, LI4, Ashi point *	0.25 mm, 40 mm	ST7: 25–30 mm Etc: n.r.	ST7: 90° Etc: n.r.	30 min	50 Hz	Microwave
Zhang (2014) [31]	20 sessions (once a day for 20 days)	TE3, ST7, SI19	0.30 mm, 25 mm	ST7, SI19	n.r	20 min	20 Hz	None
Han (2018) [32]	10 sessions (5 times a week for 2 wks)	SI19, ST7, ST6, LI4, Ashi point	0.32 mm, 40 mm	n.r.	n.r.	30 min	1.2 Hz	Extra corporeal Shock Wave
Hu (2018) [33]	14 sessions (14 days)	SI19, TE17, ST7, ST6, LI4, GB34, Ashi point *	0.25 mm, 25 mm/40 mm	n.r.	n.r.	30 min	1.45 Hz	Ultrashort wave
Ye (2019) [34]	10 sessions (5 times a week for 2 wks)	SI19, ST7, ST6, LI4, ST36	0.25 × 25 mm	n.r.	n.r.	30 min	Low frequency	Ultrashort wave

* Ashi point: acupuncture point without a specific name or definite location, the site of which is determined by tenderness or other pathological responses [36]. n.r., not reported; wks, weeks.

**Table 3:** Measurement method of outcome TER for TMD in the included studies.

Author, Year	Scale of TER	Symptoms Included in the TER Evaluation							
		Pain	Discomfort	Tenderness	Functional Activity	Opening Disorder	Mastication Disorder	Clicking with Function	Recurrence
Liu (2007) [24]	3-point scale	o	x	x	x	o	x	o	x
Liu (2010) [27]	3-point scale	o	x	x	x	o	x	o	x
Wang (2009) [25]	3-point scale	o	x	x	x	o	x	x	o
Jia (2010) [26]	5-point scale	x	x	o	o	o	o	o	x
Hu (2018) [32]	5-point scale	x	x	o	o	o	o	o	x
Bu(2011) [28]	4-point scale	o	x	o	x	o	x	x	o
Li (2011) [29]	5-point scale	o	o	x	x	o	x	o	o
Chen (2012) [30]	5-point scale	x	x	o	o	o	o	o	x
Zhang (2014) [31]	3-point scale	o	x	o	x	o	x	x	x
Han (2018) [32]	5-point scale	o	x	o	x	o	o	o	x

(o) Symptoms are included in the TER evaluation. (x) Symptoms are not included in the TER evaluation. TER, total effectiveness rate; TMD, temporomandibular disorder.

**Table 4:** Risk of bias assessment.

First Author, Year	Selection Bias		Performance Bias	Detection Bias	Attrition Bias	Reporting Bias
	Random Sequence Generation	Allocation Concealment	Blinding of Participants	Blinding of Outcome Assessment	Incomplete Outcome Data	Selective Reporting
Liu (2007) [24]	H	U	H	L	L	U
Wang (2009) [25]	U	U	H	U	L	U
Jia (2010) [26]	U	U	H	U	L	U
Liu (2010) [27]	U	U	H	U	L	U
Bu (2011) [28]	U	U	H	U	L	U
Li (2011) [29]	U	U	H	U	L	U
Chen (2012) [30]	U	U	H	U	L	U
Zhang (2014) [31]	U	U	H	U	L	U
Han (2018) [32]	U	U	H	U	L	U
Hu (2018) [33]	L	U	H	U	L	U
Ye (2019) [34]	H	U	H	U	L	U

H, high risk; L, low risk; U, unclear risk.

## Data Availability

The datasets used and/or analyzed during the current study are available from the corresponding author upon reasonable request.
